# Dissolution Behaviour of Metal-Oxide Nanomaterials in Various Biological Media

**DOI:** 10.3390/nano13010026

**Published:** 2022-12-21

**Authors:** Mary-Luyza Avramescu, Marc Chénier, Suzanne Beauchemin, Pat Rasmussen

**Affiliations:** 1 Environmental Health Science and Research Bureau, HECS Branch, Health Canada, 251 Sir Frederick Banting Driveway, Ottawa, ON K1A 0K9, Canada; 2 Department of Earth and Environmental Sciences, University of Ottawa, 140 Louis Pasteur, Ottawa, ON K1N 6N5, Canada

**Keywords:** nanoparticles, zinc oxide, manganese oxide, cerium oxide, aluminium oxide, iron oxide, PSF and Gamble, ICP-MS, inhalation pathway

## Abstract

Toxicological effects of metal-oxide-engineered nanomaterials (ENMs) are closely related to their distinct physical–chemical properties, especially solubility and surface reactivity. The present study used five metal-oxide ENMs (ZnO, MnO_2_, CeO_2_, Al_2_O_3_, and Fe_2_O_3_) to investigate how various biologically relevant media influenced dissolution behaviour. In both water and cell culture medium (DMEM), the metal-oxide ENMs were more soluble than their bulk analogues, with the exception that bulk-MnO_2_ was slightly more soluble in water than nano-MnO_2_ and Fe_2_O_3_ displayed negligible solubility across all tested media (regardless of particle size). Lowering the initial concentration (10 mg/L vs. 100 mg/L) significantly increased the relative solubility (% of total concentration) of nano-ZnO and nano-MnO_2_ in both water and DMEM. Nano-Al_2_O_3_ and nano-CeO_2_ were impacted differently by the two media (significantly higher % solubility at 10 mg/L in DMEM vs. water). Further evaluation of simulated interstitial lung fluid (Gamble’s solution) and phagolysosomal simulant fluid (PSF) showed that the selection of aqueous media significantly affected agglomeration and dissolution behaviour. The solubility of all investigated ENMs was significantly higher in DMEM (pH = 7.4) compared to Gamble’s (pH 7.4), attributable to the presence of amino acids and proteins in DMEM. All ENMs showed low solubility in Gamble’s (pH = 7.4) compared with PSF (pH = 4.5), attributable to the difference in pH. These observations are relevant to nanotoxicology as increased nanomaterial solubility also affects toxicity. The results demonstrated that, for the purpose of grouping and read-across efforts, the dissolution behaviour of metal-oxide ENMs should be evaluated using aqueous media representative of the exposure pathway being considered.

## 1. Introduction

Due to their enhanced physical–chemical properties (chemical, optical, electrical, and magnetic), metal-oxide (MeOx)-engineered nanomaterials (ENMs) have been increasingly used in different industries, including pharmaceuticals and personal care products (https://www.mordorintelligence.com/industry-reports/metal-oxide-nanoparticles-market). Among the most abundantly produced ENMs worldwide are CeO_2_, FeOx, AlOx, and ZnO (100 to 1000 t/year, [1]) due to their broad applications. ZnO ENMs are widely used in sunscreens, cosmetics, and antimicrobial agents, while CeO_2_ ENMs are used in catalyst, fuel cell industries, UV-coatings, absorbents, and paints [2]. MnO_2_, Fe_2_O_3_, and Al_2_O_3_ ENMs have applications in biomedicine, medical diagnosis, and therapeutics [1,3]. Al_2_O_3_ ENMs are also used in the catalyst industry and wastewater treatment [4]. Due to their electrochemical and oxidative properties, MnO_2_ ENMs have various applications in cancer therapy, as magnetic resonance imaging (MRI) contrast agents, in biosensors and battery production, soil remediation, and in industrial wastewater treatment [2,5].

The worldwide increase in production and application of ENMs has raised concerns about possible effects of human exposure via oral and inhalation pathways [5,6,7]. Currently, these concerns are being addressed through international hazard assessment efforts which focus on grouping and read-across strategies to reduce reliance on animal testing [8,9]. Toxicological effects of ENMs, including MeOx, are closely related to their distinct physical–chemical properties [10], out of which solubility and surface reactivity are recognised to be particularly important for read-across justification [8]. In nanomedicine applications, knowledge of ENM dissolution in biological fluids is also important as biopersistent ENMs may pose “potential long-term toxicity to internal tissues/organs” [11,12]. Efforts are thus made worldwide to develop inorganic therapeutic agents that are safe and degradable under physiological conditions’ effects [11,12,13,14,15] as this may contribute to a reduction of long-term accumulation in the body and mitigate toxicity.

Solubility is used as a screening criterion in the hazard assessment of ENMs as it is a key physicochemical characteristic that affects their biopersistence and biokinetic behaviour [6,9,16,17,18]. Consequently, solubility data are particularly valuable for grouping ENMs for hazard assessment [9,19,20]. Based on the percentage of water solubility as the screening criterion, OECD 2015/44 [19] divided ENMs into four categorie: high (>70%), moderate (10–70%), low (1–10%), and negligible (<1%) solubility. Similarly, a water solubility threshold of 100 mg/L was used by the DF4nanoGrouping initiative for human inhalation toxicity [9,20] to classify ENMs as soluble (group 1) or biopersistent (groups 2, 3, and 4). Alternatively, Oberdorster and Kuhlbusch [6] proposed prioritizing studies that compare the dissolution of metal compounds in physiologically relevant fluids over those focused on water solubility. Park et al. [21] proposed an x-fold algorithm to facilitate the similarity assessment of two ENMs by quantifying differences in key physicochemical properties determining human hazards. Applying these approaches, Keller at al. [22] assessed pairwise similarity between silica nanoforms by quantifying differences in dissolution rates (in simulated lung fluids) to aid read-across and grouping for a specific nanoform. Previous grouping and read-across strategies had been based on determining whether water solubility of an ENM is similar to that of its bulk analogue or that of another nanoform [23]. Avramescu et al. [24] cautioned that bulk-CuO would not be a good model for nano-CuO due to the large differences in solubility in cell culture media (38×) compared to water (6×).

The purpose of the present study was to investigate the dissolution behaviour of five raw (uncoated) metal-oxide ENM powders (ZnO, MnO_2_, CeO_2_, Al_2_O_3_, and Fe_2_O_3_) in water and cell culture medium at initial ENM concentrations relevant for toxicological studies (10 and 100 mg/L). The influence of particle size was evaluated by comparing the dissolution behaviour of the five ENMs with that of their bulk analogues. In addition to water and cell culture medium, the dissolution behaviour of three ENMs (ZnO, MnO_2_, CeO_2_) was further evaluated in two simulated lung fluids to investigate how the various aqueous media influence solubility. This comparative study of the dissolution behaviour of metal-oxide ENMs in biologically relevant media will inform future grouping and read-across efforts.

## 2. Materials and Methods

### 2.1. Materials and Reagents

Uncoated metal-oxide nano-powders, including zinc(II) oxide (ZnO, 35–45 nm), manganese(VI) oxide (MnO_2_, 40–60 nm), cerium(VI) oxide (CeO_2_, 10–30 nm), aluminium(III) oxide (γ-Al_2_O_3_, <50 nm), and iron(III) oxide (α-Fe_2_O_3_, 30 nm), and their bulk powder analogues were purchased from US Research Nanomaterials, Inc. (Houston, TX, USA), Skyspring Nanomaterials, Inc. (Houston, TX, USA), and Sigma-Aldrich (Oakville, ON, Canada). Metal-oxide characteristics provided by suppliers are presented in Table S1 (Supplementary Materials). The nanomaterials were selected for their relevance to metal-oxide ENMs currently in commerce in Canada.

The crystallographic structure and purity of all metal-oxide ENMs was confirmed by powder X-ray diffraction using a Rigaku Ultima IV Diffractometer (University of Ottawa X-ray facility), and the results are summarised in Table S2 (Supplementary Materials) and detailed elsewhere [25].

Ultrapure water (Milli-Q 18.2 MΩ cm) was used for the preparation of all metal-oxide dispersions, solutions, and reagents. Certified single- and multi-element standard solutions (1000 μg/mL) from Delta Scientific Ltd. (Mississauga, ON, Canada) were used to prepare matrix-matched calibration standards. Soluble Al, Ce, Fe, Mn, and Zn salts (Sigma-Aldrich, Oakville, ON, Canada) were used to prepare solutions to evaluate recovery during dissolution experiments from each media. Low- and high-level trace elements in water-certified reference materials (TM-24.4 and TMDA 64.3) used for analytical quality assessment were purchased from Environment Canada (Ottawa, ON, Canada).

The four media used to investigate dissolution of metal-oxides were: ultrapure water, cell culture medium, Gamble’s fluid, and phagolysosomal simulant fluid (PSF).

The cell culture medium used in the study was Dulbecco’s Modified Eagle’s Medium (DMEM; pH = 7.4) Nutrient Mixture F-12HAM (Life Technologies Inc., Burlington, ON, Canada), supplemented with 2% foetal bovine serum (FBS), 45 IU/mL penicillin, and 45 IU/mL streptomycin. The composition and preparation of DMEM cell culture medium was described by Avramescu et al. [24], based on Decan et al. [26].

The two simulated lung fluids (SLF) used in this study—Gamble’s solution and phagolysosomal simulant fluid (PSF)—represent different interstitial conditions in the lung/respiratory tract, as recommended by ISO/TR19057:2017 [16]. The two SLFs are salt solutions with different pH levels (typically pH 7 for Gamble’s and pH 4.5 for PSF), consisting of inorganic salts (carbonates, chlorides, sulphates, and phosphates) and organic compounds (citrate, acetate, glycine), with Gamble’s solution favouring the formation of insoluble complexes due to neutral pH and the presence of carbonates [27].

The preparation of PSF was conducted according to Stefaniak et al. [28], as recommended by ISO/TR-19057/2017. The PSF solution was used to model dissolution in lung alveolar macrophages [28,29], and this fluid is buffered with 0.02 M potassium hydrogen phthalate. After preparation, the pH of the fluid was adjusted to 4.55 (±0.1) with 1N KOH [28]. The alkylbenzyldimethylammonium chloride (ABCD) was omitted from PSF preparation to avoid potential interferences, as recommended by Stefaniak et al. [29]. The PSF fluid has been used to study nanomaterial dissolution by other authors [30,31,32] and was also found to be consistent with *in vitro* clearance [30,32].

The Gamble’s solution was prepared as recommended by ISO/TR19057:2017 [16] and described by Marques et al. [33], Stebounova et al. [34], and Midander et al. [35]. After preparation, the media pH was adjusted to 7.4 (±0.1) with 2N HCl and further to pH 7.22–7.25 before the experiment set-up to allow pH to be maintained during the experiments. Over the duration of the experiment, a maximum increase in pH of 0.1 units and 0.8 units for PSF and Gamble’s solution, respectively, was deemed acceptable [35]. To prevent potential cross-contamination and pH drifting over time (due to contact of fluids with air), all extractions were conducted in sealed tubes [35,36,37]. The compositions of both PSF and Gamble’s media are detailed in Table S3 (Supplementary Materials). For comparing simulated lung fluids in the present study, a 24 h extraction time was selected as it was deemed adequate for the assessment of metal dissolution by other authors [35,38,39,40]. A decrease in repeatability has been observed in some fluids, including Gamble, for a longer contact time (e.g., 168 h; Henderson et al. [40]), which was attributed to metal complexation and precipitation, and difficulties in maintaining the pH level.

### 2.2. Particle Size Distribution and ZP Characterisation

Stock dispersions of each MeOx ENM were prepared in water and sonicated at each material-specific delivered sonication energy (DSE), as previously optimised [25]. The optimisations are presented in Table S4 (Supplementary Materials). A Zetasizer Nano ZSP (Malvern Panalytical, Westborough, MA, USA) was used to measure the particle size by dynamic light scattering (DLS) and zeta potential (ZP) by electrophoretic light scattering (ELS). These measurements were performed on each ENM stock dispersion after sonication at material-specific DSE, as previously described [24]. For DLS and ELS measurements, all ENM dispersions were sampled in triplicate. For each replicate, at least three consecutive measurements (DLS/ELS) were performed and averaged, as previously described by Avramescu et al. [24]. All particle characterisation results (DLS and ELS) are provided in the Supplementary Materials (Table S5A,B and Figure S1). It was noted that agglomeration in both PSF and Gamble dispersions yielded an exponential increase in the hydrodynamic diameter (Dh in micron range, Figure S1). Thus, ENM dispersions using PSF and Gamble were not suitable for DLS measurements due to instability and poly-dispersity.

### 2.3. Dissolution Experiments

Dissolution experiments in water, cell culture media, Gamble’s solution, and PSF were conducted using a batch protocol approach previously applied to MeOx ENMs dissolution [24] following the OECD Test Guideline 105:1995 [41]. MeOx ENM stock dispersions (prepared as described earlier) were diluted to the required initial concentration: 10 or 100 mg/L for water and DMEM and 100 mg/L for PSF and Gamble’s. Samples along with procedural blanks and spiked matrix blanks (1–10 mg/L of Al, Ce, Fe, Mn, and Zn prepared from soluble salts) were incubated in a MaxQ4000 orbital shaker (Thermo Scientific, Canada) at 37 °C for 48 h (24 h for PSF and Gamble), with occasional shaking at 100 rpm (1 h/day). Triplicate samples were taken at each incubation time and used for particle size and ZP measurements and for dissolved metal fractions’ separation and quantification, as described below. Sequential centrifugation was performed at 20,000× *g*, as described by Avramescu et al. [24], and the separation times used for MeOx experiments are presented in Table 1 along with other experimental details. The absence of particles from the resulting supernatant was confirmed by DLS [24,25]. Dissolved metal concentrations in final extracts were analysed using an Inductively Coupled Plasma Optical Emission Spectrophotometer (ICP-OES) and matrix match calibration standards. Limits of detection are provided in Table S6 (Supplementary Materials). Dissolution results were expressed as mg/L of metal dissolved and the percent of total metal concentrations. A Seven Compact S220 pH meter (Mettler Toledo) was used to monitor the pH of the samples at each incubation time and the results are presented in Tables S5A and S10 (Supplementary Materials).

### 2.4. Quality Assurance

In parallel to nanomaterial experiments, control experiments with soluble salts of Zn, Mn, Ce, Al, and Fe (spiked matrix blanks) were performed for all media to monitor the potential loss by sedimentation (Table S7A, Supplementary Materials). Generally, good spike recoveries (within 80–106%) were obtained for all media and elements at 0 h of incubation (Table S7A, Supplementary Materials). A few exceptions (e.g., less than 80% recovery for Fe, Al, Zn, and Ce in certain media) were observed after separation and will be discussed later (in Section 4.3).

Recovery for trace element reference materials (TM 24.4 and TMDA 64.3) was in the range of 96–123% for five elements (Mn, Al, Fe, Mn, and Zn, except Zn is not certified in TM 24.4). As cerium is not contained in either of those reference materials, analytical controls made from soluble salt were prepared at different concentrations and the obtained recoveries were 100.0–110.3%.

### 2.5. Statistical Analysis

One-way analysis of variance (ANOVA) or non-parametric Kruskal–Wallis ANOVA on ranks (if the assumption of normality was not met) followed by the post-hoc Tukey test for multiple comparisons were used to evaluate effects of media on metal-oxide dissolution (48 h incubation time). Student’s *t*-test or Welch’s *t*-test (if the homoscedasticity assumption was not met) were used to compare effects of particle size (nano vs. bulk) and different concentrations (10 vs. 100 mg/L) on metal-oxide dissolution (48 h incubations). The statistical significance was set at *p* < 0.05. Statistical analyses were performed using Sigma Plot v 13 and Excel (Analysis ToolPak). A summary of the statistical test results is presented in the Supplementary Materials (Table S9).

## 3. Results

The term “solubility” is used in this paper to describe measurements expressed as either mg/L or % solubility. While solubility data are illustrated as relative solubility (% of total concentration) in the figures, both units of measurement are included in tables and/or in the Supplementary Materials. Unless otherwise specified, both metrics are considered in the interpretation.

### 3.1. Effect of Particle Size on Dissolution of Five Metal-Oxides (Nano vs. Bulk)

**Figure 1:**
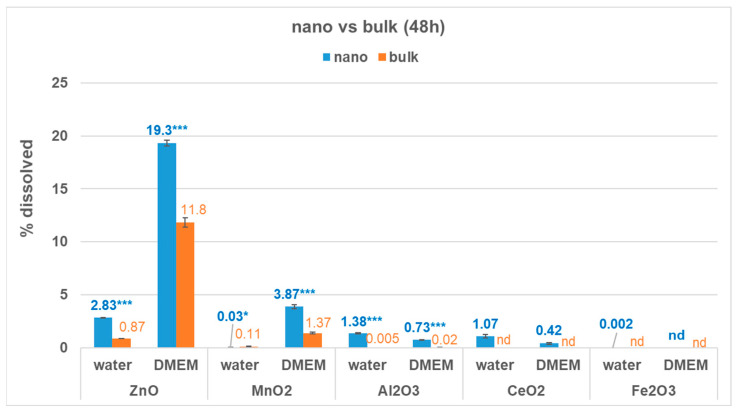
Influence of particle size (nano vs. bulk) on % solubility of MnO_2_, ZnO, CeO_2_, Al_2_O_3_, and Fe_2_O_3_ after 48 h of incubation in water and DMEM. Initial metal-oxide concentration was 100 mg/L. Results presented as mean (standard deviation) of triplicates. All pH values are reported in Tables S5A and S10, and Table S8 (Supplementary Materials) shows additional information. nd = Not detected. For a given metal-oxide and medium, ‘*’ and ‘***’ indicate significant differences between nano vs. bulk at α = 0.05 and 0.001, respectively, based on the Student’s or Welch’s *t*-test.

Two media, water and DMEM, were selected to evaluate the influence of particle size on the dissolution behaviour of five metal-oxide ENMs (ZnO, MnO_2_, CeO_2_, Al_2_O_3_, and Fe_2_O_3_) and their bulk analogues. Materials were incubated in each media using the same initial metal-oxide concentration (100 mg/L). Figure 1 presents the effect of particle size on the percent solubility of ZnO, MnO_2_, CeO_2_, Al_2_O_3_, and Fe_2_O_3_ after 48 h of incubation in water and DMEM. Additional information is presented in Table S8 (Supplementary Materials).

Results showed that, in both media, metal-oxide ENMs were more soluble than their bulk analogues (*p* < 0.001), with two exceptions. Bulk-MnO_2_ was slightly more soluble in water than nano-MnO_2_ (*p* = 0.025), and Fe_2_O_3_ was insoluble in both media regardless of particle size (nano-Fe_2_O_3_ had a negligible % solubility in water, 0.002%). However, dissolution was below 1% for both exceptions, which for grouping exercises corresponds to “negligible solubility, <1%”. In this paper, above 70% indicates high solubility, 10–70% indicates moderate solubility, 1–10% indicates low solubility, and below 1% indicates negligible solubility [19].

After 48 h, Figure 1 shows that % solubility of ENMs in water varied from low for ZnO, Al_2_O_3_, and CeO_2_ (1.07–2.83%), to negligible for MnO_2_ (0.03%). In contrast, % solubility of bulk oxides in water was either negligible (<1%, ZnO, MnO_2_, Al_2_O_3_) or not detected (CeO_2_, Fe_2_O_3_). The measured % solubility in water was barely detectable for bulk-Al_2_O_3_ (≤0.005%), while for bulk-ZnO and bulk-MnO_2_ it ranged from 0.87% to 0.11%, respectively.

Compared to water, the solubility of ENMs in DMEM after 48 h increased to moderate for nano-ZnO (19.3%/15.5 mg/L) and low for nano-MnO_2_ (3.87%/2.44 mg/L), while solubility decreased to negligible for both nano-Al_2_O_3_ (0.73%/0.39 mg/L) and nano-CeO_2_ (0.43%/0.34 mg/L) (Figure 1, Table S8). Nano-Fe_2_O_3_ solubility was barely detectable (0.002%/0.0014 mg/L) in water and was not detected in DMEM, showing that this nanomaterial is insoluble in both media. Results for the bulk analogues were similar, in that bulk solubility in DMEM increased for ZnO and MnO_2_ to moderate (ZnO, 11.8%/9.3 mg/L) and low (MnO_2_, 1.37%/0.94 mg/L), while for the other bulk materials it was either negligible (Al_2_O_3_, 0.02%/0.012 mg/L) or not detected (CeO_2_, Fe_2_O_3_).

The greatest difference between nano and bulk metal-oxide % solubility (at 48 h) was observed in water for nano-Al_2_O_3_ (271× higher than bulk), followed by nano-ZnO (3.3× higher than bulk). Similar nano vs. bulk trends were observed in DMEM, but the differences were less pronounced (36× higher for nano-Al_2_O_3_ than bulk and 1.6× higher for nano-ZnO than bulk). Interestingly, in water, nano-MnO_2_ showed a 4.4× lower % solubility than bulk, but in DMEM its % solubility was 2.8× higher than that of its bulk analogue. Nano-CeO_2_ was more soluble in water than in DMEM (Figure 1), while bulk-CeO_2_ was insoluble in both media (and therefore ratios could not be calculated for nano vs. bulk CeO_2_).

### 3.2. Effect of Initial Metal-Oxide ENM Concentration on Dissolution

**Figure 2:**
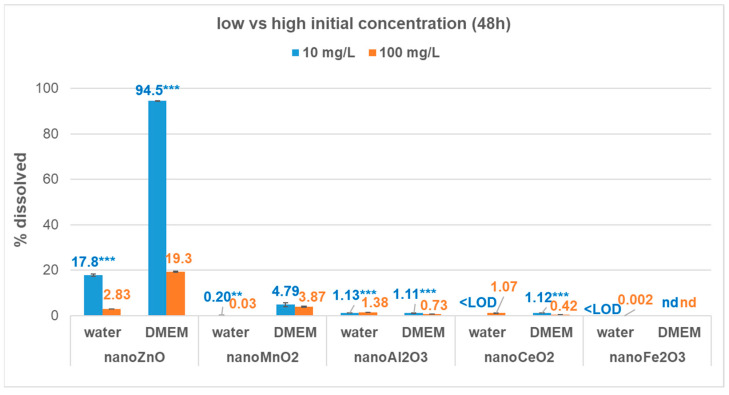
Influence of initial concentrations (10 mg/L vs. 100 mg/L) on % solubility of the ZnO, MnO_2_, CeO_2_, Al_2_O_3_, and Fe_2_O_3_ ENMs in water and DMEM after 48 h of incubation (<LOD = below the limit of detection; nd = not detected). Results are presented as mean (standard deviation) of triplicates, and pH values are reported in Table S5A. For a given metal-oxide and medium, ‘**’ and ‘***’ indicate significant differences between 10 vs. 100 mg/L at α = 0.01 and 0.001, respectively, based on the Student’s *t*-test. See Figure S2 in the Supplementary Materials for absolute mass dissolved in water and DMEM.

The dissolution behaviour of all five metal-oxide nanomaterials was evaluated in both water and cell culture medium (DMEM) at two initial concentrations: 10 mg/L (low) and 100 mg/L (high). Figure 2 presents the % solubility of ZnO, MnO_2_, CeO_2_, Al_2_O_3_, and Fe_2_O_3_ ENMs at these different initial concentrations (10 mg/L vs. 100 mg/L) after 48 h of incubation.

Figure 2 shows that, in DMEM, % solubility of all ENMs was greater at a low compared to a high initial concentration, except for Fe_2_O_3_, for which no dissolution was observed at either initial concentration. Regardless of the initial concentration, nano-Fe_2_O_3_ was insoluble in both media (Figure 2). Table S8 (Supplementary Materials) provides additional information. Overall, except for nano-ZnO, for which the relative solubility ranged from 2.83% to 94.5%, the % solubility of MeOx ENMs was low to negligible (4.79% to <1%) in both media.

In water, both nano-ZnO and nano-MnO_2_ had significantly higher % solubility at a low initial concentration (*p* < 0.001 and *p* = 0.009, respectively, Figure 2). However, the dissolution of nano-ZnO was greater in DMEM compared to water at both starting concentrations. The trend was similar for nano-MnO_2_, with increased dissolution in DMEM compared to water. The initial concentration had a varying effect on the relative magnitude of ZnO and MnO_2_ ENM % solubility in both water and DMEM. In water, the greatest difference between a low and high initial concentration was observed for nano-MnO_2_ (7.9× higher at 10 mg/L than at 100 mg/L), followed by nano-ZnO (6.3× higher). The difference in relative solubility between a low and high initial concentration was less pronounced in DMEM than in water for both nano-ZnO (4.9× higher at 10 mg/L than at 100 mg/L) and nano-MnO2 (1.2× higher).

In contrast, nano-Al_2_O_3_ and nano-CeO_2_ showed a reversal in % solubility trends between the two media. At 48 h, nano-Al_2_O_3_ and nano-CeO_2_ displayed significantly greater % solubility at a lower initial concentration in DMEM (*p* ≤ 0.001), but a reverse trend was observed in water (*p* < 0.001, Figure 2). Nano-Al_2_O_3_ had 1.5× higher % solubility at a low vs. high initial concentration in DMEM and 1.2× higher % solubility at a high vs. low initial concentration in water. Similarly, nano-CeO_2_ displayed 2.7× higher % solubility in DMEM at a low vs. high initial concentration, but in water its solubility was measurable only at the high initial concentration. Thus, an overall observation was that dissolution in both water and DMEM varied with the type of MeOx ENM and the initial concentration.

### 3.3. Effect of Aqueous Media on Dissolution of Metal-Oxide ENMs

The influence of aqueous media on % solubility was evaluated by comparing the dissolution behaviour of three metal-oxide nanomaterials (nano-ZnO, nano-MnO_2_, and nano-CeO_2_) in four different aqueous media (PSF, Gamble, DMEM, and water). The Al_2_O_3_ and Fe_2_O_3_ were not considered for further investigation with Gamble and PSF due to time limitations and observed loss by sedimentation in DMEM (discussed later, Section 3.4) and/or negligible solubility in both water and DMEM.

**Figure 3:**
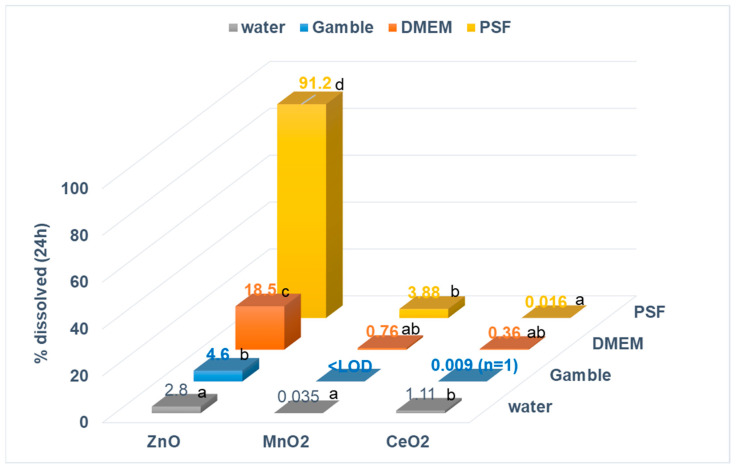
Influence of aqueous medium on % solubility of nano metal-oxides at a 100 mg/L initial concentration (<LOD = below the limit of detection). Extraction time was 24 h (grey, water; blue, Gamble; red, DMEM; yellow, PSF), and pH = 4.5 for PFS, 7.4 for DMEM and Gamble, and 7.8, 6.3, and 4.6 for ZnO, MnO_2_, and CeO_2_, respectively, in water. For a given metal-oxide, values followed by the same letter are not statistically different at α = 0.05. See Table S8 for standard deviations and Table S9B for results of statistical tests.

Figure 3 shows the influence of an aqueous medium on the % solubility of the three ENMs after 24 h of incubation in PSF, Gamble, DMEM, and water at an initial metal-oxide concentration of 100 mg/L. Results showed that MeOx ENM % solubility varied with the investigated media and was strongly dependent on the investigated material (Figure 3). The greatest dissolution was observed in PSF for both nano-ZnO and nano-MnO_2_, but in water for nano-CeO_2_. Nano-ZnO displayed almost complete dissolution in PSF (91.2%), which was significantly higher (*p* < 0.001) compared to other aqueous media (Figure 3). In other media, the % solubility of nano-ZnO varied from moderate in DMEM (18.5%) to low in Gamble (4.6%) and water (2.8%). Similarly, nano-MnO_2_ % solubility was increased in PSF compared to other media, where % solubility was either negligible (<1%, DMEM and water) or not observed (Gamble, <LOD). However, a Tukey test showed that while nano-MnO_2_ dissolution was significantly higher in PSF (*p* = 0.020) compared to water, there was no statistically significant difference in its dissolution behaviour with DMEM (*p* > 0.05).

In contrast to nano-ZnO and nano-MnO_2_, nano-CeO_2_ showed a significantly increased relative solubility (*p* = 0.020) in water (1.11%) compared to PSF (<0.016%). In all other media tested (Figure 3), nano-CeO_2_’s solubility was negligible (<1%) and differences between DMEM and PSF were not statistically significant (*p* = 0.372). In Gamble, nano-MnO_2_ and nano-CeO_2_ showed negligible solubility and they were not included in statistical comparisons for those materials. All statistical test results are summarised in the Supplementary Materials (Table S9B).

Overall, all ENMs investigated showed low solubility in Gamble media (pH = 7.4) compared with PSF (pH = 4.5), and % solubility of all ENMs was significantly higher (*p* < 0.001) in DMEM compared to Gamble (Figure 3). Additionally, agglomeration in both PSF and Gamble dispersions yielded an exponential increase in particle size, resulting in Dh values in the micron range (Figure S1, Supplementary Materials). Further research would be needed to determine the extent to which the increased agglomeration observed in PSF and Gamble dispersions influenced ENM solubility.

### 3.4. Evidence of Losses by Sedimentation during Control Experiments with Soluble Salts

**Figure 4:**
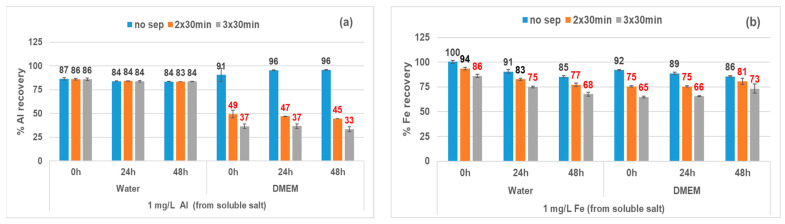
Evidence of Al (**a**) and Fe (**b**) sedimentation during control experiments with soluble salts: analyte loss increases as centrifugation time increases. Results (% recovered in supernatant) are presented as mean (standard deviation) of triplicates. no sep = No centrifugation, 2 × 30 min and 3 × 30 min centrifugation at 20,000× *g*.

Generally, evidence of loss by sedimentation was not observed during control experiments performed with soluble salts, with the following exceptions: Al in DMEM, Fe in DMEM and water, Ce in PSF and Gamble, and Zn in Gamble. Experiments with soluble salts of Al and Fe are shown in Figure 4. There was evidence of losses of Al due to sedimentation as the centrifugation time increased (24 and 48 h, Figure 4a) for DMEM (up to 50% after 24 h and 67% after 48 h) but not for water. Figure 4b presents a similar loss of Fe in both DMEM (up to 25% after 24 h and 35% after 48 h) and in water (more pronounced after 48 h). While not observed in water or DMEM, evidence of Ce sedimentation was observed in both PSF (97% loss) and Gamble (95% loss) media after 24 h (Table S7B, Supplementary Materials). In Gamble, Zn losses due to sedimentation were observed after 24 h, but the magnitude was lower (39% loss).

## 4. Discussion

### 4.1. Effect of Particle Size (Nano vs. Bulk) and Initial ENM Concentration on Dissolution

Overall, ZnO, CeO_2_, and Al_2_O_3_ ENMs were more soluble than their bulk analogues in both water and DMEM (Figure 1), and these results are in agreement with our previous study on dissolution of nano- vs. bulk-CuO [24]. One exception was nano-MnO_2_ in water, which was less soluble than bulk-MnO_2_ in water. Fe_2_O_3_ was insoluble regardless of particle size and media.

The pH is an important factor that influences dissolution, and proton-promoted dissolution is commonly observed for metal-oxide ENMs [42,43,44,45,46,47]. In this study, the nano/bulk pH ratio for ZnO, MnO_2_, and Al_2_O_3_ dispersions in water varied from 0.93 to 1.01, indicating that pH alone cannot explain the observed differences in solubility between the nano and bulk materials. Along with pH, particle size was found to be an important factor affecting the solubility of ZnO nanoparticles [42,48], with ZnO nanoparticles having an increased solubility and initial dissolution rate compared with bulk-ZnO at various pH levels [42].

In the case of CeO_2_, the pH of the nano-dispersion in water was 4.54, compared to 6.43 for bulk-CeO_2_ dispersion (Tables S5A and S10). Along with particle size, this difference in pH may contribute to the increased solubility of CeO_2_ ENM in water compared to its bulk analogue since Ce(IV) is more soluble at pH < 5 [44,49,50]. The increased solubility of CeO_2_ ENM in water observed in our study may also be related to the occurrence of exchangeable Ce(III) at the surface of CeO_2_ nanoparticles [44]. Ce(III) was found at the surface of CeO_2_ nanoparticles in concentrations increasing with a decrease in the particle size [51,52]. In DMEM, CeO_2_ ENM had a low % solubility, but its bulk analogue was insoluble (Figure 1). No difference in pH was observed between the nano- and bulk-CeO_2_ dispersions in this medium (1.02 ratio nano/bulk). Therefore, particle size rather than pH appears to be the main contributor to the difference in solubility between CeO_2_ materials in DMEM. In addition, CeO_2_ ENM % solubility was lower in DMEM compared to water (Figure 1). This decreased solubility may be related to the presence of phosphate in the DMEM [44,45,53,54,55]. Phosphate can inhibit the dissolution of CeO_2_ ENM through either retention of P on the CeO_2_ surfaces or formation of Ce(III)PO_4_ surface precipitates which are associated with charge reversal [44]. This inhibiting effect would be supported by our results, which showed that ZP was positive in water (43.8 mV) but negative in DMEM (−13.2 mV).

Nano-MnO_2_ was the exception in that it showed a lower % solubility in water compared to its bulk analogue (Figure 1), and this was observed not only at 48 h but also at 24 h (Supplementary Materials, Table S8). The DLS results show that this is not caused by separation interferences (Table S5A). The pH of MnO_2_ ENM dispersion in water was similar to that of bulk-MnO_2_ (nano/bulk pH ratio 0.93). Both the surface charge and the high specific surface area of this highly amorphous MnO_2_ ENM (XRD analysis, Table S2) may favour adsorption of released ions on NPs, resulting in lower dissolved ions compared to bulk-MnO_2_ [2,56]. The MnO_2_ ENM dispersion in water showed a negative ZP (−30.2 mV), whereas the ZP of other MeOx ENM dispersions in water was positive (Table S5A). This observation is in agreement with other studies [57] that also reported negative ZP for 2D MnO_2_ in water. A more negative surface charge increases the adsorption of positive cations [56].

In addition to particle size, the initial concentration had a pronounced influence on the dissolution of ZnO ENM in both water and DMEM, while the differences were less pronounced for MnO_2_, Al_2_O_3_, and CeO_2_ ENMs (Figure 2). Fe_2_O_3_ was insoluble in both media regardless of the initial concentration tested. The relative solubility of ZnO ENM was enhanced in both media at a lower initial concentration, but the magnitude was higher in DMEM. In contrast, CeO_2_ and Al_2_O_3_ ENMs showed an increased % solubility at a low initial concentration in DMEM, but the reverse trend in water (Figure 2).

This pronounced increase of ZnO ENM solubility in DMEM compared to water may be related to the presence of FBS in the media. The proteins/serum and organic compounds (e.g., acids, cysteine) present in biological media were found to stimulate dissolution of ZnO nanoparticles, either by complexing the ion released from the surface or by ligand-enhanced dissolution [58,59,60,61]. At a low (10 mg/L) initial concentration, ZnO ENM dissolution was almost complete in DMEM (92%), showing that at this concentration the cell cultures will be exposed mainly to dissolved species. For the hazard assessment, this indicates that the focus should be on dissolved species rather than particles in the case of this ENM. This observation is relevant for the interpretation of toxicity results as increased ENM solubility also affects toxicity [62,63,64]. While less pronounced, this effect of increased % solubility with a decreasing initial concentration was also observed for MnO_2_, CeO_2_, and Al_2_O_3_ ENM in DMEM (Figure 2). In case of those ENMs, cell cultures will be exposed not only to particles but also to small amounts of dissolved species when effects are evaluated at low ENM concentrations.

However, this effect of increased % solubility with a decreasing initial concentration was not observed in DMEM for all ENMs, despite the enhanced solubility observed in this medium. As we observed in our previous study [24], CuO ENM % solubility was increased at a higher compared to a lower initial concentration (51.5% vs. 12.6%, respectively). These results show that different media-dependent mechanisms may influence the dissolution behaviour of each ENM, and for the purpose of hazard assessment, it is important to evaluate the dissolution of ENMs at concentrations relevant to toxicity assays.

### 4.2. Effect of Aqueous Media on Dissolution of Metal-Oxide ENMs

The extent to which a property of ENM affects hazards depends not only on the particularities of the ENM but also on those of the exposure route [21]. That means, materials with different properties (e.g., shape, solubility) may result in different toxic effects, depending on the specific exposure route being considered (e.g., inhalation vs. dermal). Dissolution in biologically relevant media (e.g., for inhalation exposure) was proposed as a criterion under Tier 2 for grouping ENMs [DF4nanoGrouping, 9,20]. Gamble and PSF media are considered representative of inhalation pathways. The first simulates the near-neutral lung-lining fluid, and the second mimics the acidic fluid that inhaled particles are exposed to after phagocytosis by alveolar macrophages [16,33]. The complexity of biologically relevant media (e.g., simulated lung fluids or cell culture media) may influence dissolution results due to the interaction of the dissolved fraction with specific media components [65,66,67] by increasing or inhibiting dissolution. The presence of ligands such as phosphate was shown to inhibit the dissolution of nano-MnO_2_ [45] and nano-CeO_2_ [44,55]. In contrast, small organic ligands such as biologically relevant carboxylic acids (e.g., lactic, citrate, malic, succinic, acetic, glutaric, ascorbic acids) were found to enhance the release of ions from CeO_2_ NPs to varying extents, at pH 4.5 [49,50,68].

In the present study, we assessed dissolution of three ENMs (ZnO, MnO_2_, and CeO_2_) after 24 h of incubation using simulated lung fluids (PSF and Gamble) along with DMEM and water (initial metal-oxide concentration of 100 mg/L). Our results showed that the solubility of all investigated ENMs varied with the media used (Figure 3, Table S8). Among all ENMs tested, nano-ZnO showed the highest variation with media, as its solubility increased from low in water and Gamble (2.83%/2.28 mg/L and 4.62%/3.71 mg/L, respectively), to moderate in DMEM (18.5%/14.9 mg/L) and high in PSF (91.2%/73.3 mg/L). While less pronounced, the % solubility of MnO_2_ and CeO_2_ ENMs varied from low to negligible depending on the medium (Figure 3).

All ENMs tested displayed increased dissolution in PSF compared to Gamble (Figure 3) due to the higher hydrogen ion concentration in PSF [42,43,44,46]. These results are in agreement with previous studies [22,69,70]. The low solubility of ZnO ENM in near-neutral Gamble (4.6%, 3.71 mg/L) observed in our study may be indicative of biodurability and accumulation in the interstitial lung environment. However, it’s very high solubility (91.2%, 73.3 mg/L) in acidic PSF media (pH = 4.5) may indicate potential bioavailability and transformation inside the lysosomes. The latter observation suggests that dissolution may be the main process defining this ENM’s fate in the lungs, with released ions rather than undissolved particles causing the observed effects. Holmfred et al. [69] observed a rapid 10-fold higher solubility in PSF (exceeding the nominal dose of 102 mg/L) compared to low-calcium Gamble medium for ZnO ENM (NM-110). The dissolution half-time of ZnO nanoparticles in PSF media was found to be short (t_1/2_ < 1 h), suggesting that clearance is dominated by dissolution and effects from released ions for this ENM [22]. Uski et al. [71] observed that ZnO NPs (crystalline size 56 nm) were internalised into the cells (macrophage cell line RAW 264.7) and induced cell cycle arrest and cytotoxicity after phagocytosis due to released Zn(II) ions by dissolution inside lysosomes.

Our results indicate durability (i.e., negligible solubility) for CeO_2_ ENM in both simulated lung fluids; however, its solubility was slightly increased in PSF compared to Gamble (Figure 3, Table S8). This was also reported by Holmfred et al. [70], who observed measurable dissolution of CeO_2_ (NM-112) in PSF (0.029 × 10^−3^ mg/L/h) but not in low-Ca Gamble medium. Based on its slow dissolution (t_1/2_ > 1 year) in PSF, Keller et al. [22] suggested that CeO_2_ nanoparticle effects will be dominated by the particles, and therefore, accumulation may be of concern, which is also supported by our findings. Li et al. [72] observed increased pH-dependent dissolution of rare-earth oxide nanoparticles (including CeO_2_) after “macrophage uptake and lysosomal processing”, followed by depletion of lysosomal phosphate due to complexation with released Ce ions and deposition on NP surfaces. Moreover, *in vivo* and *in vitro* studies showed that Ce ion speciation changed inside the lung, with a predominance of Ce(III) ions inside cells vs. outside cells [73,74]. Consequently, the behaviour of CeO_2_ ENM in biological systems may be defined by dissolution and reprecipitation. Considering that within 24 h after inhalation almost 90% of inhaled particles will be phagocytised by macrophages (Aladova et al., 2007 cited by Innes et al., 2021 [75]), it is important to assess ENM dissolution at pH 4.5 to better characterise its bioaccessibility and durability in the lung [75]. Based on our results, the fate of the tested ENMs in the lungs may be determined by bioaccessibility for ZnO, by durability for CeO_2_, and possibly by a combination of both for MnO_2_.

Differences in aqueous media have important effects on dissolution, not only due to differences in pH but also due to differences in media composition (e.g., presence of complexing agents). The chemical composition of media (with similar pH) had an impact on the mobilisation or immobilisation of metals from the MeOx ENMs investigated, affecting the dissolution results. DMEM and Gamble media have a similar pH (7.4) but different compositions [57,76,77,78]. Compared to Gamble, DMEM contains different ligands (HO-, Cl-, amino acids, FBS) that can interact with metals and promote dissolution via the formation of soluble inorganic and organic complexes [58,59,60,61,78,79,80,81]. For all three MeOx ENMs, higher dissolution was observed in DMEM than in Gamble media (Figure 3).

In our study, MnO_2_ ENM dissolution was measurable in DMEM but not in Gamble (Figure 3), and this may be due to the complex mix of 21 amino acids present in DMEM that can act as either chelating or reducing agents, enhancing dissolution. Gray et al. [57] observed depletion of various amino acids (e.g., tyrosine, tryptophan, methionine, lysine, histidine, and arginine) in the cell medium after exposure to MnO_2_ materials, which is consistent with reductive dissolution in cell culture medium of MnO_2_ due to redox reactions with various “weak reducing agents”.

We also observed enhanced dissolution of CeO_2_ ENM in DMEM compared to Gamble, likely due to the interaction of reactive ligand groups present in DMEM with Ce ions and/or reductive dissolution. In contrast to Gamble, DMEM contains iron compounds (e.g., FeSO_4_). In the presence of Fe(II), the Ce(IV) can be reduced to Ce(III), which enhances the dissolution of CeO_2_ nanoparticles [82,83]. Schwabe et al. [45] observed that the presence of strong chelating agents can form complexes with Ce(III), promoting its stabilisation in solution.

### 4.3. Evidence of Losses by Sedimentation during Control Experiments with Soluble Salts

In this study, centrifugation was selected as the separation method to avoid artefacts caused by ultrafiltration [25,30,65], but the results (Section 3.4 and Figure 4) showed that media-related sedimentation artefacts are difficult to completely avoid. Consequently, the dissolved fraction measured for those specific combinations of analyte and media may be an underestimation of solubility; that is, an “apparent solubility” that represents the fraction not matrix-bound under the experimental conditions employed in the study. For example, released Al can interact either with proteins (their phosphate cofactor [66]) or with phosphate in the media [67], followed by aluminium phosphate precipitation, which may account for the observed losses by sedimentation. Similarly, in the case of nano-CeO_2_ in PSF and Gamble, losses may be related to the presence of ligands, as phosphate has been shown to inhibit the dissolution of nano-CeO_2_ by complexation with Ce ions, followed by precipitation [44,55].

Fe_2_O_3_ was insoluble in DMEM regardless of particle size. Control experiments with Fe-soluble salts showed that losses of Fe spike occurred in water as well as in DMEM (Figure 4b), which differed from the behaviour of the Al spike (Figure 4a). Consequently, different mechanisms may be responsible for the observed loss of Fe in water, which was not observed for other metals.

In general, losses of analyte by sedimentation appears to be related to complexation of the dissolved fraction by specific media components [65,66,67]. Further research would be needed to identify the precise mechanism(s) causing these analyte losses.

### 4.4. Implications for Grouping and Read-Across

Table 2 presents groupings of MeOx ENMs and their bulk analogues based on dissolution results in water and DMEM (48 h of incubation, 100 mg/L initial concentration) using screening criteria/categorisation approaches from OECD ENV/JM/MONO (2015)44 [19]. Results from our current study (MnO_2_, ZnO, CeO_2_, Al_2_O_3_, Fe_2_O_3_) are presented along with previous results (CuO, NiO, TiO_2_) from Avramescu et al. [24]. Depending on the category for the purpose of the hazard assessment, the focus can be only on particles (“negligible” category), on particles but “taking into account small amounts of dissolved species” (“low” category), both particles and dissolved species (“moderate” category), or only dissolution species (“high” category). Solubility is important for hazard assessment since it affects biopersistence and biokinetics, in that increased ENM dissolution contributes to the lung clearance by decreasing the overall lung clearance rate [6,9,16,17,18].

Using this classification, distinct differences emerged between nano and bulk solubility of some MeOx, as well as their solubility in water vs. DMEM (Table 2). Based on % solubility in water, MeOx ENMs were classified in two categories, “low” (ZnO, CeO_2_, Al_2_O_3_, NiO) and “negligible” (MnO_2_, Fe_2_O_3_, CuO, TiO_2_), while all their bulk analogues were classified as “negligible (<1%)”. The dissolution behaviour of bulk analogues in water indicates that effects are dominated by particles. Consequently, in the context of read-across, bulk analogues will not be good models for ENMs based on their dissolution in water (at a 100 mg/L initial concentration). However, this is not the case if dissolution in DMEM is considered, as most ENMs are grouped in the same category with their bulk analogue, except for CuO and NiO ENMs (Table 2). Our results indicate that the aqueous medium is an important consideration in the context of read-across from bulk- to nano-scale metal-oxides, as categorisation based on % solubility in water and DMEM did not agree. However, in DMEM, the results at a lower initial concentration (Figure 2) indicate that for accurate interpretation of the effects, dissolved species are important to consider in the case of ZnO, CeO_2_, and Al_2_O_3_ ENMs, especially for ZnO ENM which dissolved almost completely at a low concentration but only partially at a high concentration.

We also evaluated the impact of aqueous media on grouping MeOx ENMs based on dissolution results in PSF, Gamble, DMEM, and water using the same criteria approach [19] (Table 3). Dissolution in biologically relevant media was proposed as a criterion under Tier 2 for grouping ENMs [DF4nanoGrouping initiative, 9,20]. Again, amongst all the ENMs tested, ZnO ENM showed the highest variation (Table 3), as it was classified into three different categories: “low” (water and Gamble), “moderate” (DMEM), and “high” (PSF). While the effect of media on grouping of the other ENMs was not that pronounced, it was nevertheless observed (MnO_2_, CeO_2_, Table 3). Consequently, dissolution tests in biologically relevant media representative of the exposure pathway will not only help categorisation and read-across efforts but will also aid in the design of toxicity assays. For the hazard assessment, evaluating the dissolution behaviour of ENMs at concentrations relevant to toxicity assays will facilitate better interpretation/correlation with adverse effects.

However, grouping of MeOx ENMs cannot be based solely on dissolution results, and further studies are required to validate the use of other properties (e.g., surface reactivity, *in vitro* toxicity) for grouping and read-across [8]. Nevertheless, our results showed that assessing dissolution in relevant media at concentrations representative of the exposure pathway being assessed is important in the context of grouping and read-across from ENM to bulk analogues and from one ENM to another.

## 5. Conclusions

The results of this study showed that, for the purpose of human health hazard assessment, the dissolution behaviour of metal-oxide ENMs should be evaluated using aqueous media that are representative of the exposure pathway being considered. Out of the five metal-oxides investigated, nano-ZnO was most influenced by the aqueous medium, and its % solubility ranged from “high” (in PSF), to “moderate” (in DMEM), to “low” (in both water and Gamble’s solution) when evaluated at a 100 mg/L initial concentration. The least affected ENM was Fe_2_O_3_, which displayed negligible solubility across all tested aqueous media.

However, nano-ZnO dissolution in DMEM showed a concentration-dependent effect that would place it in the “high” category when evaluated at a 10 mg/L initial concentration. Other studied ENMs (nano-CeO_2_ and nano-Al_2_O_3_) also displayed contrasting dissolution trends in DMEM according to the initial concentration (i.e., increased % solubility with decreasing initial concentration). Based on their dissolution behaviour in DMEM, both nano-CeO_2_ and nano-Al_2_O_3_ would be classified as having a “low” % solubility when evaluated at an initial concentration of 10 mg/L, but “negligible” when evaluated at a 100 mg/L initial concentration.

These results demonstrated that the initial concentration is an important factor to be considered when preparing ENM dispersions for toxicological assays. For example, in the case of nano-ZnO at a low initial concentration (10 mg/L) in DMEM, the metal will be present almost exclusively as the dissolved fraction due to rapid dissolution, while at a high concentration (100 mg/L), both nanoparticles and dissolved metal will be present.

Finally, analyte losses due to sedimentation may occur in solubility experiments using metal-oxide ENMs, whether centrifugation or ultrafiltration is used for separation. Spike recoveries should always be tested using the soluble salt to identify this artefact, as such losses are dependent on the specific combination of ENM and aqueous medium.

## Figures and Tables

**Table 1:** Dissolved metal separation details for each metal-oxide ENM and media. Times used for undissolved particle separation by sequential centrifugation at 20,000× *g* (60 min/90 min represent 2×/3× 30 min) and element wavelengths used for dissolved metal fraction quantification for ICP-OES analysis.

Media	Time	n	ZnO		MnO_2_		CeO_2_		Al_2_O_3_		Fe_2_O_3_	
			100 mg/L	10 mg/L	100 mg/L	10 mg/L	100 mg/L	10 mg/L	100 mg/L	10 mg/L	100 mg/L	10 mg/L
Water	0 h	3	90 min	90 min	90 min	90 min	90 min	90 min	90 min	90 min	90 min	90 min
pH = 6.4 ± 0.5	24 h	3										
	48 h	3										
DMEM + 2% FBS	0 h	3	90 min	90 min	90 min	90 min	90 min	90 min	60 min	60 min	90 min	90 min
pH = 7.6 ± 0.1	24 h	3										
	48 h	3										
PSF	0 h	3	60 min		60 min		90 min					
pH = 4.5 ± 0.02	24 h	3										
Gamble	0 h	3	60 min		60 min		60 min					
pH = 7.5 ± 0.1	24 h	3										
Element, wavelength (ICP-OES)			Zn 213.857 nm		Mn 257.610 nm		Ce 418.659 nm		Al 396.152 nm		Fe 238.204 nm	

**Table 2:** Grouping of MeOx ENMs and their bulk analogues based on dissolution results in water and DMEM (48 h of incubation, 100 mg/L initial concentration) using screening criteria from OECD ENV/JM/MONO (2015)44. Results from the current study (MnO_2_, ZnO, CeO_2_, Al_2_O_3_, Fe_2_O_3_) combined with results from Avramescu et al. [2020] (CuO, NiO, TiO_2_). In blue are the ones that fall in a different category (“negligible”) at 24 h than at 48 h. Red boxes show nano falling in a different category than bulk (nd = not detected; <LOD = below the limit of detection); * 24 h results (48 h not available).

Media	Form (Initial Concentration)	MeOx	High (>70%)	Moderate (10–70%)	Low (1–10%)	Negligible (<1%)
Water	Nano (100 ppm)	ZnO			ZnO, 2.83%* (2.28 mg/L)*	
		MnO_2_				MnO_2_, 0.03%* (0.02 mg/L)*
		CeO_2_			CeO_2_, 1.07%* (0.87 mg/L)*	
		Al_2_O_3_			Al_2_O_3_, 1.38%* (0.73 mg/L)*	
		Fe_2_O_3_				Fe_2_O_3_, <0.01%* (<10 ug/L)*
		CuO *				CuO, 0.99% (0.79 *mg/L*)
		NiO			NiO, 1.22%* (0.96 mg/L)*	
		TiO_2_ *				TiO_2_, <0.01%* (<10 ug/L)*
	Bulk (100 ppm)	ZnO				ZnO, 0.87%* (0.70 mg/L)*
		MnO_2_				MnO_2_, 0.11%* (0.08 mg/L)*
		CeO_2_				CeO_2_, nd
		Al_2_O_3_				Al_2_O_3_, <0.01%* (<10 ug/L)*
		Fe_2_O_3_				Fe_2_O_3_, <LOD
		CuO *				CuO, 0.17%* (0.19 mg/L)*
		NiO				NiO, 0.05%* (0.05 mg/L)*
		TiO_2_ *				TiO_2_, <0.01%* (<1 ug/L)*
DMEM	Nano (100 ppm)	ZnO		ZnO, 19.3%* (15.5 mg/L)*		
		MnO_2_			MnO_2_, 3.87%* (2.44 mg/L)*	
		CeO_2_				CeO_2_, 0.42%* (0.34 mg/L)*
		Al_2_O_3_				Al2O_3_, 0.73%* (0.39 mg/L)*
		Fe_2_O_3_				Fe_2_O_3_, nd
		CuO		CuO, 51.5%* (41.1 mg/L)*		
		NiO			NiO, 1.81%* (1.42 mg/L)*	
		TiO_2_ *				TiO_2_, 0.04%* (0.03 mg/L)*
	Bulk (100 ppm)	ZnO		ZnO, 11.8%* (9.8 mg/L)*		
		MnO_2_			MnO_2_, 1.37%* (0.94 mg/L)*	
		CeO_2_				CeO_2_, nd
		Al_2_O_3_				Al_2_O_3_, 0.02%* (0.01 mg/L)*
		Fe_2_O_3_				Fe_2_O_3_, nd
		CuO			CuO, 1.51%* (1.39 mg/L)*	
		NiO				NiO, 0.07%* (0.06 mg/L)*
		TiO_2_ *				TiO_2_, <0.01%* (<1 ug/L)*

* 24 h results (48 h not available).

**Table 3:** Grouping of MeOx ENMs based on dissolution results in PSF, Gamble, DMEM, and water (24 h of incubation, 100 mg/L initial concentration) using the screening criteria/categorisation approach from OECD ENV/JM/MONO (2015)44. (<LOD = below the limit of detection).

MeOx (Initial Concentration)	Media	High (>70%)	Moderate (10–70%)	Low (1–10%)	Negligible (<1%)
nano-ZnO (100 ppm)	Water			ZnO, 2.83%* (2.27 mg/L)*	
	DMEM		ZnO, 18.5%* (14.9 mg/L)*		
	Gamble			ZnO, 4.62%* (3.71 mg/L)*	
	PSF	ZnO, 91.2%* (73.3 mg/L)*			
nano-MnO_2_ (100 ppm)	Water				MnO_2_, 0.03%* (0.02 mg/L)*
	DMEM				MnO_2_, 0.76%* (0.48 mg/L)*
	Gamble				MnO_2_, <LOD
	PSF			MnO_2_, 3.88%* (2.45 mg/L)*	
nano-CeO_2_ (100 ppm)	Water			CeO_2_, 1.11%* (0.90 mg/L)*	
	DMEM				CeO_2_, 0.36%* (0.29 mg/L)*
	Gamble				CeO_2_, 0.01%* (<0.01 mg/L)*
	PSF				CeO_2_, 0.02%* (0.01 mg/L)*

## Data Availability

Research data are not shared.

## Supplementary Materials

The following supporting information can be downloaded at: https://www.mdpi.com/article/10.3390/nano13010026/s1, Table S1: Characteristics of nano and bulk metal-oxides as detailed in the supplier’s certificate of analysis or website. Table S2: Crystallographic structure and purity of metal-oxide ENMs confirmed by powder X-ray diffraction. Table S3: Chemical composition (g/L) of PSF and Gamble’s solutions. Table S4: Sonication details for MeOx ENM stock dispersions prepared in water. Table S5A: Characterisation of nano metal-oxide dispersions at different incubation times using dynamic light scattering (DLS) and electrophoretic light scattering (ELS). Table S5B: Characterisation of metal-oxide ENM stock dispersion (water) before and after sonication using dynamic light scattering (DLS) and electrophoretic light scattering (ELS). Table S6: Zn, Mn, Ce, Al, and Fe limits of detection obtained for water, DMEM, PSF, and Gamble’s fluids. Table S7A: Recoveries of soluble salts (0 h) for all media and elements. Table S7B: Soluble salts’ recoveries (0–48 h) for PSF, Gamble, and elements. Table S8: Metal-oxide dissolution in water, DMEM + 2% FBS, and Gamble’s and PSF fluids. Table S9: Statistical test results. Table S10: pH of bulk metal-oxide dispersions at different incubation times in water and DMEM. Figure S1: Hydrodynamic diameter of agglomerates vs. % solubility of ENMs dispersed in four aqueous media; Figure S2. Influence of initial concentrations (10 mg/L vs. 100 mg/L) on the absolute mass dissolved in water and DMEM after 48-h incubation of the ZnO, MnO_2_, CeO_2_, Al_2_O_3_, Fe_2_O_3_ ENMs. (< LOD = below limit of detection; nd = not detected). Results presented as mean (standard deviation) of triplicates. Plus eight references.
