# Supplementary material for: Dissolution Behaviour of Metal-Oxide Nanomaterials in Various Biological Media
Source: Nanomaterials (Basel). 2022 Dec 21;13(1):26. doi: 10.3390/nano13010026 (PMC9824292; doi:10.3390/nano13010026)
Supplement: Supplementary file 1 [file nanomaterials-13-00026-s001.zip › nanomaterials-2010518-supplementary.pdf]

nanomaterials

Table S1. Characteristics of nano and bulk metal oxides as detailed in supplier's certificate of analysis or website.

Material	Description	CAS #	Average particle size (nm)	Specific surface area (SSA, m2/g)	Purity	Color
nano-Al_2O_3 [a]	γ-Aluminum Oxide Nanopowder	1344-28-1	< 50	> 40 **	not specif	White powder
nano-CeO_2 [b]	Cerium oxide Nanopowder	1306-38-3	10-30	30-50	99.97% (REO)	light yellow powder
nano-Fe_2O_3 [b]	α-Iron (III) Oxide Nanopowder	1309-37-1	30	20-60	99.5+%	red brown powder
nano-MnO_2 [c*]	Manganese (IV) Oxide Nanopowder	1317-34-6	40-60	~13.5	98+%	Black powder
nano-ZnO [b]	Zinc Oxide Nanopowder	1314-13-2	35-45	~65 **	99+%	Milky white powder
bulk-Al_2O_3 [c]	γ-Aluminum Oxide Bulk powder	1344-28-1	1030	1.41	99.90%	White powder
bulk-CeO_2 [c]	Cerium Oxide Bulk powder	1306-38-3	~5000		99.9+% (REO), 99% (TREO)	pale yellow-white powder
bulk-Fe_2O_3 [b]	α-Iron (III) Oxide Bulk powder	1309-37-1	5000	***	99.9%	
bulk-MnO_2 [b]	Manganese (IV) Oxide Bulk powder	1313-13-9	~5000 (D50); <10000 (D90)		99+%	Black powder
bulk-ZnO [c]	Zinc Oxide Bulk powder	1314-13-2	1000	5.8	>99.9+%	Milky white powder

a Sigma-Aldrich (Oakeville, Canada)

b US Research Nanomaterials, Inc. (Houston, TX, USA)

c Skyspring Nanomaterials, Inc. (, USA)

* sold as Manganese Oxide (Mn_2O_3) Powder but identified as MnO_2 by XRD (Avramescu et al 2019)

** SSA (BET) from Bushell et al 2020: nAl_2O_3 = 129 m2/g; nanoZnO: nZnO=35 m^2/g

*** manufacturer contacted but this is not available

Table S2. Crystallographic structure and purity of metal oxide ENMs confirmed by powder X-ray diffraction using a Rigaku Ultima IV Diffractometer (University of Ottawa X-ray facility). Results are detailed in Avramescu et al. 2019 (Nanosafe).

Nanomaterial	XRD results	SAXS results
nano-Al_2O_3	95% tetragonal Alumina gamma (boehmite-derived), $(Al_2O_3)_{1.333}$ with 5% hexagonal corundum, Al_2O_3; crystallinity 19.1 (4), highly amorphous; nanocrystal sizes 4.0 (4) nm (γ-Al_2O_3) and 42.6 (16) nm (corundum)	Two distinct size distributions 8.1 nm (28.5 % RSD) and 26.7 (3.2 % RSD) nm representing 89.2% and 10.8% volume % of the sample. Compared with XRD results may correspond to γ-Al_2O_3 and corundum, respectively. Both particle shapes = spheroid.
nano-CeO_2	>99% cubic Cerianite-(Ce), syn; crystal size 9.4 (1.4) nm; crystallinity 55.4 (3)	
nano-Fe_2O_3	>99% hexagonal synthetic hematite; crystal size 14.3 (0.5) nm; crystallinity 43.6 (5).	
nano-MnO_2	Highly amorphous MnO_2 (not Mn_2O_3 as in manufacturer specif.) 78.1 % hexagonal Aktenskite, crystal size 20.1 (16.3) nm; crystallinity 15.7 (3). 21.9% orthorhombic Ramsdellite, crystal size 32.2nm; crystallinity 14.7 (3)	
nano-ZnO	>99% hexagonal zinc oxide, crystal size 16.3 (1.2) nm; crystallinity 71.3 (17)	

Table S3. Chemical composition of PSF and Gamble solutions (g/L) as recommended by ISO/TR19057 (2017) and described in Stefaniak et al 2005 (PSF) and Stebounova et al 2011, Marques et al. 2011, Moss 1979 (Gamble).

Order of addition	Chemical	Formula	PSF	Gamble
		pH	4.5 ± 0.1	7.4
1	Magnesium Chloride	$MgCl_2*6H_2O$	-	0.203
2	Sodium Chloride	NaCl	6.65	6.019
3	Potassium Chloride	KCl	-	0.298
4	Sodium Phosphate dibasic anh.	Na_2HPO4	0.142	0.142
5	Sodium Sulphate anh.	Na_2SO_4	0.071	0.071
6	Calcium Chloride dihydrate	$CaCl_2*2H2O$	0.029	0.368
7	Sodium Acetate	$C_2H_3O_2Na$	-	0.953
8	Sodium hydrogen carbonate	$NaHCO_3*3H_2O$	-	2.604
9	Sodium Citrate dihyrate *(representative of proteins)*	$C_6H_5Na_3O_7*2H_2O$	-	0.097
10	Sodium Hydroxide	NaOH	-	-
11	Citric Acid	$C_6H_8O_7$	-	-
12	Glycine *(representative of organic acids)*	H_2NCH_2COOH	0.45	-
13	SodiumTartrate dihyrate	$C_4H_4O_6Na_2*2H_2O$	-	-
14	Sodium Lactate	$C_3H_5NaO_3$	-	-
15	Sodium Pyruvate	$C_3H_3O_3Na$	-	-
16	Formaldehide			
17	Potassium Hydrogen Phtalate	$1(HO_2C)-2-(CO_2K)-C_6H_4$	4.085	-

Note: Full chemical composition of the DMEM media as described in Avramescu et al. 2020, Electronic Supplementary Material: Table S5 Full composition of the DMEM/F-12 no phenol red (Life technologies, cat # 21041) as found on supplier website (https://www.thermofisher.com/ca/en/home/technical-resources/media-formulation.57.html).

Table S4. Sonication details for MeOx ENM stock dispersions prepared in water. Material specific delivered sonication energy (DSE) previously optimized (Avramescu et al 2019), except for the necessity to repeat the optimization when equipment was updated.

MeOx	Setting *	Time (min)	P(W)	DSE (J/mL)
nano-Mn_2O_3	40%	5	39.1	117
nano-CeO_2	60%	12	65.1	469
nano-Fe_2O_3	55%	10	56.4	338
nano-Al_2O_3	55%	30	54.1	974
nano-ZnO	40%	14	32	269

* pulse (8s ON, 2s OFF)

Table S5A. Characterization of nano metal oxide dispersions at different incubation times using dynamic light scattering (DLS) and electrophoretic light scattering (ELS). Mean and standard deviation (SD) of three replicates.

ENM	Media	initial conc.	Time	n	Z-Ave d.nm		PdI		Pk 1 Mean Int d.nm		Pk 1 Area Int %		ZP (mV)		pH	
		MeOx			mean	SD	mean	SD	mean	SD	mean	SD	mean	SD	mean	SD
nano-Al_2O_3	Water	10 mg/L	0h	3	404	51	0.49	0.04	216	14	92.8	1.3	44.7	2.5	6.31	0.03
			24h	3	454	36	0.51	0.02	217	17	94.9	3.2	39.2	3.5	6.47	0.22
			48h	3	460	124	0.57	0.11	197	18	97.7	2.1	43.8	3.1	6.40	0.21
		100 mg/L	0h	3	282	28	0.42	0.04	306	26	88.3	6.7	46.0	1.2	6.21	0.11
			24h	3	372	11	0.46	0.01	286	6	89.8	0.6	47.0	2.8	6.14	0.07
			48h	3	380	44	0.54	0.07	411	124	73.5	17.1	46.2	1.1	6.31	0.11
	DMEM	10 mg/L	0h	3	237	18	0.64	0.14	344	54	81.2	1.2	-11.7	0.3	7.88	0.03
			24h	3	280	31	0.57	0.13	282	17	84.3	1.6	-12.6	0.4	7.97	0.03
			48h	3	523	124	0.63	0.07	424	37	86.1	8.9	-13.2	0.5	7.88	0.07
		100 mg/L	0h	3	349	9	0.44	0.02	331	13	95.3	1.5	-14.1	0.3	7.86	0.01
			24h	3	375	19	0.43	0.01	322	17	96.1	1.6	-12.5	0.4	7.86	0.06
			48h	3	312	45	0.41	0.04	332	23	96.4	3.9	-10.9	1.2	7.86	0.03
nano-CeO_2	Water	10mg/L	0h	3	391	92	0.50	0.07	163	8	92.8	7.8	10.3	3.2	5.44	0.07
			24h	3	278	117	0.40	0.11	146	26	97.9	2.6	2.9	0.6	5.37	0.04
			48h	3	508	205	0.55	0.13	118	10	98.8	0.2	-5.7	5.1	5.96	0.06
		100mg/L	0h	3	245	11	0.38	0.02	192	8	95.8	1.7	37.6	2.6	4.84	0.20
			24h	3	196	47	0.39	0.17	226	50	94.9	4.2	43.8	1.5	4.58	0.12
			48h	3	232	34	0.39	0.02	197	15	96.6	0.4	44.1	1.5	4.54	0.01
	DMEM	10mg/L	0h	3	398	84	0.56	0.11	136	12	100.0	0.0	-11.3	0.2	7.90	0.03
			24h	3	295	41	0.42	0.04	161	15	97.8	2.4	-9.7	0.7	7.81	0.08
			48h	3	338	41	0.46	0.05	187	8	96.6	4.0	-8.4	0.3	7.72	0.04
		100mg/L	0h	3	268	29	0.41	0.03	218	15	96.1	1.7	-12.2	1.0	7.94	0.09
			24h	3	176	6	0.22	0.01	205	10	99.0	0.4	-13.2	0.2	8.02	0.05
			48h	3	162	3	0.21	0.01	193	8	99.3	0.2	-11.5	1.2	7.78	0.03
	Gamble	100 mg/L	0h	3	1137	63	0.42	0.08	850	175	99.3	0.9	-3.4	0.6	7.88	0.04
			24h	3	1422	175	0.50	0.11	918	234	100	0	-3.8	0.5	7.97	0.04
	PSF	100 mg/L	0h	3	833	42	0.45	0.01	568	11	100	0	-18.5	0.4	4.52	0.01
			24h	3	883	22	0.44	0.13	621	120	100	0	-18.7	0.7	4.57	0.01
nano-Fe_2O_3	Water	10 mg/L	0h	3	479	67	0.54	0.02	134	11	93.1	4.3	35.1	7.6	6.22	0.16
			24h	3	389	100	0.45	0.08	101	8	100.0	0.0	21.3	2.7	6.16	0.25
			48h	3	314	82	0.42	0.09	128	12	98.9	1.3	25.1	3.4	6.38	0.15
		100 mg/L	0h	3	198	30	0.42	0.04	191	14	75.8	9.9	39.8	1.9	6.23	0.15
			24h	3	204	32	0.40	0.00	204	25	89.3	13.7	28.8	0.9	6.33	0.18
			48h	6	183	65	0.27	0.10	169	25	91.9	11.9	36.0	5.7	6.37	0.17
	DMEM	10 mg/L	0h	3	311	46	0.45	0.05	195	17	98.9	0.5	-10.6	0.5	8.07	0.03
			24h	3	288	36	0.39	0.01	186	18	97.2	0.5	-9.8	0.3	7.91	0.06
			48h	3	309	35	0.42	0.04	203	39	93.8	7.0	-9.0	0.4	7.78	0.02
		100 mg/L	0h	3	256	45	0.44	0.04	328	91	93.3	2.2	-11.1	0.4	7.97	0.03
			24h	3	255	23	0.40	0.02	224	32	93.0	5.3	-12.9	0.6	8.05	0.09
			48h	3	255	27	0.45	0.06	221	14	83.2	14.8	-12.8	0.3	7.96	0.06
nano-MnO_2	Water	10 mg/L	0h	3	182	5	0.28	0.01	120	3	100	0	-22.6	4.8	6.22	0.47
			24h	2	192	26	0.28	0.03	120	7	100	0	-16.4	3.2	6.45	0.19
			48h	3	184	5	0.29	0.01	118	1	100	0	-24.2	0.8	6.60	0.18
		100 mg/L	0h	3	146	1	0.23	0.01	157	6	99.1	0.9	-32.0	3.0	6.18	0.19
			24h	3	145	1	0.22	0.01	157	8	99.0	0.1	-28.2	6.7	6.30	0.23
			48h	3	175	54	0.28	0.04	192	88	98.0	2.5	-30.2	2.4	6.10	0.45
	DMEM	10 mg/L	0h	3	183	17	0.30	0.01	149	8	99.0	0.1	-10.1	0.2	7.94	0.06
			24h	3	250	23	0.39	0.04	182	6	100.0	0.0	-6.7	0.4		
			48h	3	553	172	0.72	0.12	174	34	100.0	0.0	-10.1	1.6		
		100 mg/L	0h	3	160	1	0.21	0.02	172	9	99.4	0.5	-10.5	0.2	7.93	0.03
			24h	3	189	4	0.26	0.02	185	12	99.2	0.3	-10.7	0.6	7.77	0.03
			48h	3	249	19	0.36	0.05	219	6	98.9	1.1	-9.6	0.6	7.66	0.01
	Gamble	100 mg/L	0h	3	549	69.6	0.37	0.05	520	134	95.4	5.8	-11.1	0.1	7.92	0.05
			24h	3	962	26.7	0.56	0.08	559	87	100.0	0	-7.3	0.6	7.94	0.07
	PSF	100 mg/L	0h	3	565	114.5	0.37	0.04	506	167	93.1	10.3	-18.2	0.5	4.50	0.02
			24h	3	1581	17.3	0.89	0.03	371	39	100.0	0	-18.3	0.8	4.51	0.01
			48h	3	1576	78.9	0.88	0.08	380	56	100.0	0	-17.0	1.4	4.56	0.03
nano-ZnO	Water	10 mg/L	0h	3	413	17	0.51	0.03	201	15	98.4	2.5	18.2	1.0	7.85	0.04
			24h	3	452	76	0.50	0.03	243	38	98.5	2.6	4.4	0.9	7.58	0.02
			48h	3	1420	459	0.74	0.35	551	527	96.4	6.2	-8.3	3.9		
		100 mg/L	0h	3	286	17	0.45	0.07	261	38	76.9	10.5	19.1	0.4	7.73	0.04
			24h	3	201	5	0.27	0.04	195	17	99.0	1.1	20.5	0.5	7.80	0.03
			48h	3	208	2	0.25	0.03	217	17	98.2	1.1	19.0	0.7	7.81	0.07
	DMEM	10 mg/L *	0h	3	729	221	0.69	0.05	65	51	51.3	10.8	-9.1	0.3	7.91	0.02
			24h	3	225	105	0.38	0.06	14.3	3.1	50.2	3.2	-10.1	0.3	7.91	0.04
			48h	3	150	52	0.29	0.05	12.7	1.1	56.9	14.1	-9.5	0.6	7.84	0.03
		100 mg/L	0h	3	406	13	0.48	0.02	359	23	90.2	4.3	-9.9	0.7	8.07	0.04
			24h	3	468	38	0.53	0.02	324	46	88.7	4.5	-10.1	1.5	8.03	0.02
			48h	3	434	33	0.53	0.04	380	80	79.3	7.4	-14.5	0.6	7.98	0.07
	Gamble	100 mg/L	0h	3	2333	200	0.33	0.07	1694	251	100.0	0	-9.1	0.7	7.80	0.05
			24h	3	3414	655	0.52	0.11	1535	217	100.0	0	-9.0	0.2	7.86	0.05
	PSF	100 mg/L *	0h	3	2951	795	0.97	0.06	155	51	100.0	0	-12.1	1.7	4.76	0.01
			24h	3	6504	4239	0.99	0.02	89	26	77.8	19.2	-8.3	1.7	4.71	0.01

DLS results informative (very low counts <300kcps with attenuator 11) since the sample is > 90% dissolved

Table S5B. Characterization of metal oxide ENM stock dispersion (water) before and after sonication using dynamic light scattering (DLS) and electrophoretic light scattering (ELS). Mean and standard deviation (SD) of replicates.

ENM	Dispersion Media	time	Z-Ave d.nm			PdI		Pk 1 Mean Int d.nm		Pk 1 Area Int %		ZP (mV)			pH		
			n	mean	SD	mean	SD	mean	SD	mean	SD	n	mean	SD	n	mean	SD
nano-ZnO	water	no sonic	17	1403	207	0.51	0.13	776	155	100.0	0.0						
		after sonic	25	263	19	0.38	0.05	275	43	91.3	7.9	24	19.5	1.7	21	7.66	0.06
nano-MnO_2	water	no sonic	4	661	69	0.59	0.10	439	348	95.2	9.7						
		after sonic	21	154	17	0.23	0.04	171	30	98.4	1.5	18	-24.2	4.4	18	6.33	0.17
nano-CeO_2	water	no sonic	3	237	29	0.39	0.04	191	14	97.5	0.7						
		after sonic	20	236	30	0.40	0.03	197	15	95.5	2.0	19	36.6	8.0	18	3.97	0.41
nano-Al_2O_3	water	no sonic	12	772	148	0.56	0.08	463	44	99.4	1.4						
		after sonic	12	338	27	0.46	0.03	324	28	87.3	4.7	12	46.2	4.3	12	6.28	0.19
nano-Fe_2O_3	water	no sonic	8	2827	3909	0.80	0.11	428	143	97.6	5.2						
		after sonic	14	178	18	0.39	0.05	192	23	88.4	8.8	14	34.6	7.3	14	7.02	0.15

A Zetasizer Nano ZSP (Malvern Panalytical, Westborough, MA,USA) was used to measure particle size by dynamic light scattering (DLS) and zeta potential (ZP) by electrophoretic light scattering (ELS). The instrument software reports summary statistics of mean hydrodynamic diameter (Dh, z-avg), poly-dispersity index (PDI) from cumulants analysis, peak diameters from frequency distribution analysis, and ZP (Malvern Instruments Ltd. 2019a, b). As recommended by the manufacturer, dust-free disposable cuvettes (DTS0012) were used for DLS measurements and disposable zeta cells (DST1070) for zeta potential measurements.

While the primary size of all MeOx ENMs was less than 60nm (Table S1), after the dispersion in water (Table S5b) the measured hydrodynamic diameter (154-263 nm) and the first particle size distribution peak (171-275nm) of all materials were in the same order of magnitude, except for nano-Al_2O_3 that showed higher values (338nm and 324nm respectively). For some materials, the sonication was not that efficient at lower concentration (ex. CeO_2, Fe_2O_3). The zeta potential of MeOx ENMs dispersions in water was positive (19.5-46.2mV), except for nano-MnO_2 dispersion that showed a negative ZP (-24.2mV). This is in agreement with other studies (Gray et al 2021) that also measured negative ZP for 2D MnO_2 in water.

All MeOx ENMs when diluted in DMEM generally maintained the same order of magnitude of particle size (hydrodynamic diameter and the first particle size distribution peak, table S5A) in spite of the high-salt content of the media that promotes aggregation. This may be due to absorption on nanoparticles of proteins present in DMEM (e.g. FBS) that is known to decrease aggregation. (Xu and Grassian 2017) In contrast, dilutions in PSF and Gamble media showed high values (micrometer range) and variability of DLS size results (both cumulants and distribution analysis) indicating that those dispersions are not suitable for DLS due to increased instability and poly-dispersity. The increased ionic strength (e,g. PSF 0.14 M) of PSF and Gamble promote colloidal instability or agglomeration/aggregation of nanoparticles possibly due to ionic charge screening in this high-salt fluids (Gray et al 2018, David et al. 2012; Mudunkotuwa et al. 2012, Fuentes et al 2021, McClements et al., 2017, Ilett et al 2020, Xu and Grassian 2017, references). This is in agreement with Gray et al (2018) that also observed that PSF promotes aggregation due to high-salt content and lack of proteins that have dispersing effect, but the same is not true for cell culture media. Nano-ZnO dissolution in PSF (100 mg/L initial concentration) and DMEM (10mg/L initial concentration) was too fast to obtain good DLS data. However, in both cases the very low derived count rate at maximum attenuator (11, no attenuation) observed in DLS measurement are in agreement with high solubility measured by ICP-OES for those experiments.

Table S6. Zn, Mn, Ce, Al, Fe limits of detection obtained for water, DMEM, PSF and Gambles fluids. Detection limits calculated as three times the standard deviation of minimum 10 procedural blanks. Matrix match calibration used for each media.

Media	LOD (µg/L)				
	Zn	Mn	Ce	Al	Fe
DIW	13.0	1.64	5.31	1.47	1.22
DMEM	7.42	4.58	2.26	1.16	19.6
PSF	11.4	37.2	2.57		
Gamble	9.75	20.3	3.86		

Table S7A. Recoveries of soluble salts (0h) for all media and elements. Soluble salts solution at a 1mg/L analyte were used for control experiments (* 10mg/L Zn for water and DMEM experiments).

Element	Media	% spike recovery (0h)						separation
		**before separation**			**after separation**			at 20000g
		n	mean	SD	n	mean	SD	
Zn	water*	6	91	2.7	6	92	2.9	90min
	DMEM*	9	99	1.2	9	98	1.7	90min
	PSF	3	93	1.0	3	92	0.4	*60min*
	Gamble	3	97	0.7	3	97	0.2	*60min*
Mn	water	6	100	2.0	6	100	2.3	90min
	DMEM	6	106	3.6	6	105	2.0	90min
	PSF	3	99	0.5	3	98	1.3	*60min*
	Gamble	3	98	0.4	3	99	0.3	*60min*
Ce	water	9	97	2.6	9	97	2.1	90min
	DMEM	5	105	0.8	8	93	4.1	90min
	PSF	3	102	0.6	3	101	2.1	90min
	Gamble	3	99	1.9	3	0.7	0.0	*60min*
Al	water	6	80	7.4	6	84	2.0	90min
	DMEM	6	101	12.6	6	57	9.0	*60min*
Fe	water	6	100	1.7	6	86	1.6	90min
	DMEM	6	88	4.7	6	41	25.9	90min

Table S7B. Soluble salts recoveries (0-48h) for PSF and Gamble and elements.

Element	Media	Time	% recovery						separation
			**no sep**			**after separation**			at 20000g
			**n**	**mean**	**SD**	**n**	**mean**	**SD**	
Mn	**PSF**	0h	3	99	0.5	3	98	1.3	*60min*
		24h	3	98	1.0	3	97	0.5	*60min*
		48h	3	98	0.6	3	96	0.6	*60min*
	**Gamble**	0h	3	98	0.4	3	99	0.3	*60min*
		24h	3	98	0.4	3	98	1.1	*60min*
Zn	**PSF**	0h	3	93	1.0	3	92	0.4	*60min*
		24h	3	92	0.8	3	91	0.6	*60min*
	**Gamble**	0h	3	97	0.7	3	97	0.2	*60min*
		24h	3	79	1.1	3	61	2.4	*60min*
Ce	**PSF**	0h	3	102	0.6	3	101	2.1	90min
		24h	3	40	7.3	3	2.4	1.4	90min
	**Gamble**	0h	3	99	1.9	3	0.7	0.0	*60min*
		24h	3	97	0.6	3	4.2	1.4	*60min*

Table S8. Metal oxide dissolution in water, DMEM+2%FBS, Gamble's and PSF fluids (metal oxide initial concentration: 10 mg/L and 100 mg/L) presented as mean and standard deviation (SD) of triplicates. The results are expressed as mg/L metal released and as percent metal released.

		MeOx		DIW				DMEM				Gamble				PSF			
MeOx	Form	initial	Time	mg/L dissolved		% dissolved		mg/L dissolved		% dissolved		mg/L dissolved		% dissolved		mg/L dissolved		% dissolved	
		concentration		mean	SD	mean	SD	mean	SD	mean	SD	mean	SD	mean	SD	mean	SD	mean	SD
ZnO	Nano	10 mg/L	0h	1.33	0.02	16.4	0.23	7.92	0.01	94.0	0.12								
			24h	1.34	0.00	16.5	0.05	8.10	0.01	96.1	0.16								
			48h	1.44	0.04	17.8	0.47	7.96	0.01	94.5	0.13								
		100 mg/L	0h	2.26	0.01	2.81	0.01	11.7	0.04	14.6	0.05	1.53	0.22	1.90	0.27	73.5	0.01	91.4	0.02
			24h	2.27	0.02	2.83	0.03	14.9	0.27	18.5	0.33	3.71	0.65	4.62	0.81	73.3	0.19	91.2	0.23
			48h	2.28	0.02	2.83	0.02	15.5	0.21	19.3	0.26								
	Bulk	100 mg/L	0h	0.59	0.03	0.74	0.02	10.4	0.05	12.5	0.46								
			24h	0.62	0.01	0.76	0.01	9.84	0.07	11.7	0.54								
			48h	0.70	0.01	0.87	0.01	9.83	0.06	11.8	0.46								
MnO_2	Nano	10 mg/L	0h	0.005	0.001	0.082	0.008	<LOD		<LOD									
			24h	0.011	0.005	0.17	0.07	0.019	0.001	0.29	0.01								
			48h	0.013	0.005	0.20	0.08	0.303	0.059	4.79	0.94								
		100 mg/L	0h	0.016	0.000	0.025	0.001	0.020	0.000	0.031	0.001	0.049	0.016	0.078	0.026	<LOD		<LOD	
			24h	0.022	0.004	0.035	0.006	0.48	0.02	0.76	0.03	<LOD		<LOD		2.45	0.23	3.88	0.36
			48h	0.016	0.001	0.026	0.001	2.44	0.12	3.87	0.20								
	Bulk	100 mg/L	0h	0.048	0.005	0.072	0.005	0.013	0.002	0.020	0.004								
			24h	0.080	0.022	0.113	0.018	0.197	0.012	0.269	0.017								
			48h	0.080	0.003	0.113	0.011	0.943	0.064	1.366	0.084								
CeO_2	Nano	10 mg/L	0h	nd		nd		0.064	0.008	0.78	0.10								
			24h	nd		nd		0.072	0.012	0.88	0.15								
			48h	<LOD		<LOD		0.091	0.004	1.12	0.05								
		100 mg/L	0h*	0.74	0.17	0.91	0.20	0.51	0.18	0.62	0.22	0.0007	0.0004	0.0009	0.0005	0.035	0.000	0.043	0.000
			24h	0.90	0.16	1.11	0.20	0.29	0.014	0.36	0.02	0.007		0.009		0.013	0.001	0.016	0.001
			48h	0.87	0.12	1.07	0.15	0.34	0.068	0.42	0.08								
	Bulk	100 mg/L	0h	nd		nd		nd		nd									
			24h	nd		nd		nd		nd									
			48h	nd		nd		nd		nd									
Al_2O_3	Nano	10 mg/L	0h	0.077	0.009	1.46	0.17	0.053	0.004	1.00	0.08								
			24h	0.039	0.008	0.73	0.15	0.066	0.005	1.25	0.10								
			48h	0.060	0.001	1.13	0.02	0.059	0.005	1.11	0.09								
		100 mg/L	0h	0.22	0.04	0.42	0.07	0.49	0.021	0.93	0.04								
			24h	0.32	0.03	0.60	0.06	0.40	0.002	0.75	0.00								
			48h	0.73	0.03	1.38	0.05	0.39	0.021	0.73	0.04								
	Bulk	100 mg/L	0h	0.0032	0.0007	0.0056	0.0013	0.011	0.0018	0.020	0.0031								
			24h	0.0029	0.0001	0.0054	0.0003	0.021	0.0018	0.038	0.0023								
			48h	0.0027	0.0001	0.0051	0.0001	0.012	0.0004	0.021	0.0008								
Fe_2O_3	Nano	10 mg/L	0h	<LOD		<LOD		0.0014	0.0008	0.0205	0.012								
			24h	<LOD		<LOD		nd (bc)		nd (bc)									
			48h	<LOD		<LOD		nd (bc)		nd (bc)									
		100 mg/L	0h	0.0012	0.0005	0.0018	0.0007	nd (bc)		nd (bc)									
			24h	<LOD		<LOD		nd (bc)		nd (bc)									
			48h**	0.0014	0.0004	0.0019	0.0006	nd (bc)		nd (bc)									
	Bulk	100 mg/L	0h	<LOD		<LOD		nd (bc)		nd (bc)									
			24h	<LOD		<LOD		nd (bc)		nd (bc)									
			48h	<LOD		<LOD		nd (bc)		nd (bc)									

Notes: In blue, indicate that separation not successful as indicated by DLS.

Table S8 shows that after 24 h, similar results were observed in DMEM for nano-Al_2O_3 (20× higher than bulk), nano-MnO_2 (2.8x higher), and nano-ZnO (1.6 × higher). In water, the same trend was observed for nano-Al2O3 (111× higher than bulk) and nano-ZnO (3.7× higher than bulk), but, again, the reverse trend was observed for MnO_2 (bulk was 3.2x higher than nano). A t-test analysis indicates that the difference was significant between nano and bulk MnO_2 in water at both 24h ($p<0.001$) and 48h ($p=0.025$, *Welch test).

MeOx	comparison	Time	t-value	p values (two-tailed)	p values (one tailed)	signif. (p<0.05)	
MnO_2	nano vs bulk	24h	-7.189	0.00198	0.00099	P = <0.001	Yes (nano < bulk)
		48h*	-14.079	0.00501	0.00250	P =0.025	Yes (nano < bulk)

Table S9. Statistical tests results.

(A) Results t-test comparison size effect (nano vs bulk) and concentration effect (10 mg/L vs 100 mg/L)

comparison	MeOx	Media	t-value	p values (two-tailed)	p values (one tailed)	signif. (p<0.05)	
nano vs bulk	ZnO (48h)	Water *	140.311	7.98E-07	3.991E-07	P =<0.001	Yes (nano > bulk)
		DMEM	24.379	1.68E-05	8.40E-06	P =<0.001	Yes (nano > bulk)
	MnO_2 (48h)	Water *	-14.079	0.0050	0.0025	P =0.025	Yes (nano < bulk)
		DMEM	20.206	3.54E-05	1.77E-05	P = <0.001	Yes (nano > bulk)
	Al2O3 (48h)	Water	44.169	1.57E-06	7.86E-07	P = <0.001	Yes (nano > bulk)
		DMEM *	31.400	0.00101	0.00051	P = <0.001	Yes (nano > bulk)
10mg/L vs 100mg/L	ZnO	Water	55.044	6.52E-07	3.26E-07	P = <0.001	yes (10mg/L > 100mg/L)
		DMEM	449.764	1.47E-10	7.33E-11	P = <0.001	yes (10mg/L > 100mg/L)
	MnO_2	Water	3.817	0.0188	0.00941	P = 0.009	yes (10mg/L > 100mg/L)
		DMEM	1.676	0.169	0.0845	P = 0.085	no (10mg/L ~ 100mg/L)
	Al_2O_3	Water	-7.688	0.00154	0.00077	P = <0.001	yes (10mg/L < 100mg/L)
		DMEM	6.949	0.00225	0.00113	P = 0.001	yes (10mg/L > 100mg/L)
	CeO_2	Water	**				
		DMEM	12.219	0.00026	0.00013	P = <0.001	yes (10mg/L > 100mg/L)
	Fe_2O_3	Water	**				
		DMEM	not detected at anyof the two initial concentrations tested				
* Welch test (t-test assuming unequal variances), done with Excel (Analysis ToolPak)							
** not detected for 10mg/L experiment							

(B) Results ANOVA with post hoc Tukey test for multiple comparison media effect

**ZnO**	**One-way ANOVA**					
	Source of Variation	DF	SS	MS	F	P
	Between Groups	3	15773	5258	25495	<0.001
	Residual	8	1.65	0.206		
	Total	11	15775			
	(p<0.001, signifficant difference in mean values among the treatment groups)					
	All Pairwise Multiple Comparison (Tukey Test):					
	Comparison	Diff of Means	p	q	P	P<0.05
	PSF (ZnO) vs. water (ZnO)	88.39	4	337.1	<0.001	Yes
	PSF (ZnO) vs. Gamble (ZnO)	86.60	4	330.3	<0.001	Yes
	PSF (ZnO) vs. DMEM (ZnO)	72.60	4	276.9	<0.001	Yes
	DMEM (ZnO) vs. water (ZnO)	15.79	4	60.22	<0.001	Yes
	DMEM (ZnO) vs. Gamble (ZnO)	14.00	4	53.39	<0.001	Yes
	Gamble (ZnO) vs. water (ZnO)	1.789	4	6.825	0.006	Yes
**MnO_2**	**Kruskal-Wallis One-way ANOVA on Ranks**					
	Group	N	Median	25%	75%	
	water(MnO_2)	3	0.034	0.030	0.042	
	DMEM(MnO_2)	3	0.749	0.743	0.792	
	PSF(MnO_2)	3	3.830	3.549	4.265	
	H = 7.200 (2df) P(est.)= 0.027 P(exact)= 0.004					
	(significant difference in median values among treatment groups, p=0.004)					
	All Pairwise Multiple Comparison (Tukey Test):					
	Comparison	Diff of Ranks	q	P	P<0.05	
	PSF(MnO_2) vs water(MnO_2)	18	3.795	0.02	Yes	
	PSF(MnO_2) vs DMEM(MnO_2)	9	1.897	0.372	No	
	DMEM(MnO_2) vs water(MnO_2)	9	1.897	0.372	No	
**CeO_2**	**Kruskal-Wallis One-way ANOVA on Ranks**					
	Group	N	Median	25%	75%	
	water(CeO_2)	3	1.009	0.979	1.341	
	DMEM(CeO_2)	3	0.352	0.346	0.378	
	PSF(CeO_2)	3	0.015	0.014	0.017	
	H = 7.200 (2df) P(est.)= 0.027 P(exact)= 0.004					
	(significant difference in median values among treatment groups, p=0.004)					
	All Pairwise Multiple Comparison (Tukey Test):					
	Comparison	Diff of Ranks	q	P	P<0.05	
	water(CeO_2) vs PSF(CeO_2)	18	3.795	0.02	Yes	
	water(CeO_2) vs DMEM(CeO_2)	9	1.897	0.372	No	
	DMEM(CeO_2) vs PSF(CeO_2)	9	1.897	0.372	No	

Table S10. pH of bulk metal oxide dispersions at different incubation times in water and DMEM.

MeOx	Form	C MeOx	Time	n	pH (water)		pH (DMEM+2%FBS)	
					mean	SD	mean	SD
MnO_2	bulk	100 mg/L	0h	3	6.69	0.08	7.59	0.02
			24h	3	6.34	0.02	7.46	0.08
			48h	3	6.54	0.13	7.47	0.11
ZnO	bulk	100 mg/L	0h	3	7.86	0.06	7.83	0.02
			24h	3	7.96	0.02	7.78	0.05
			48h	3	7.92	0.03	7.70	0.07
CeO_2	bulk	100 mg/L	0h	3	6.37	0.19	7.99	0.11
			24h	3	6.54	0.17	7.76	0.03
			48h	3	6.43	0.26	7.66	0.02
Al_2O_3	bulk	100 mg/L	0h	3	6.08	0.14	7.85	0.04
			24h	3	6.15	0.16	7.88	0.02
			48h	3	6.23	0.19	7.82	0.02
Fe_2O_3	bulk	100 mg/L	0h	3	5.80	0.56	7.80	0.02
			24h	3	5.70	0.56	7.92	0.02
			48h	3	6.18	0.16	7.77	0.02

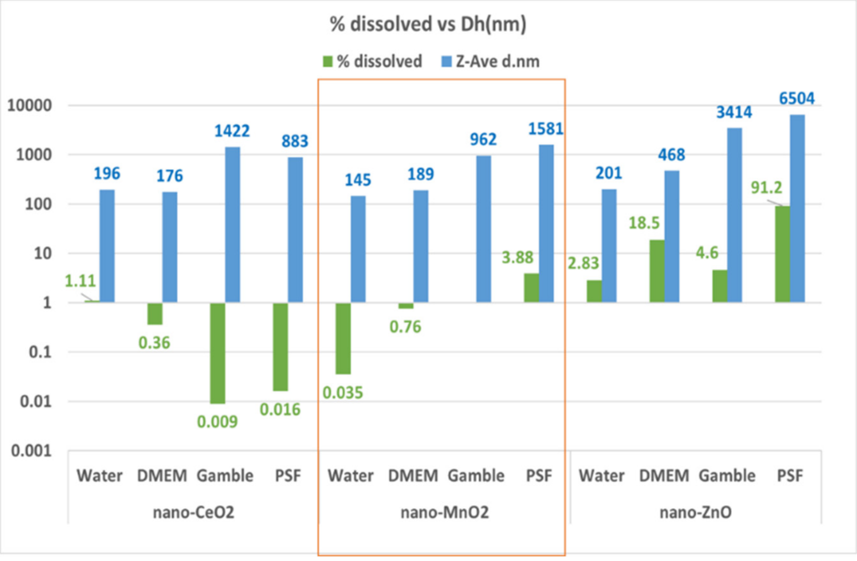

Figure S1. Hydrodynamic diameter of agglomerates versus % solubility of ENMs dispersed in four aqueous meda (water, DMEM+2%FBS, Gamble's and PSF fluids).

Agglomeration in both PSF and Gamble dispersions yielded an exponential increase in hydrodynamic diameter (Dh in micron range Fig. S1)). The results in Figure S1 indicate that dispersions using these media are not suitable for DLS measurements due to instability and polydispersity. Further research would be needed to determine the influence of increased hydrodynamic diameter associated with PSF and Gamble dispersions on ENM solubility.

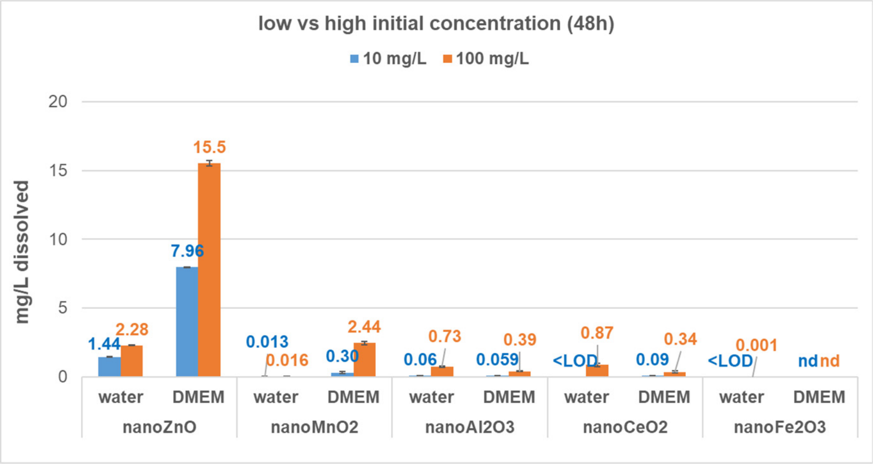

Figure S2. Influence of initial concentrations (10 mg/L vs. 100 mg/L) on the absolute mass dissolved in water and DMEM after 48-h incubation of the ZnO, MnO_2, CeO_2, Al_2O_3, Fe_2O_3 ENMs. (< LOD = below limit of detection; nd = not detected). Results presented as mean (standard deviation) of triplicates.

References

1. Fuentes C, Ruiz-Rico M, Fuentes A, Ruiz MJ, Barat JM (2020) Degradation of silica particles functionalised with essential oil components under simulated physiological conditions. J Hazard Mater 399.
2. David CA, Galceran J, Rey-Castro C, Puy J, Companys E, Salvador J, Monné J, Wallace R, Vakourov A (2012) Dissolution kinetics and solubility of ZnO nanoparticles followed by AGNES. J Phys Chem C 116:11758-11767.
3. Gray EP, Browning CL, Vaslet CA, Gion KD, Green A, Liu M, Kane AB, Hurt RH (2020) Chemical and Colloidal Dynamics of MnO2 Nanosheets in Biological Media Relevant for Nanosafety Assessment. Small 16.
4. Gray EP, Browning CL, Wang M, Gion KD, Chao EY, Koski KJ, Kane AB, Hurt RH (2018) Biodissolution and cellular response to MoO3 nanoribbons and a new framework for early hazard screening for 2D materials. Environ Sci Nano 5:2545-2559.
5. Ilett M, Matar O, Bamiduro F, Sanchez-Segado S, Brydson R, Brown A, Hondow N (2020) Nanoparticle corona artefacts derived from specimen preparation of particle suspensions. Sci Rep 10.
6. McClements DJ, Xiao H, Demokritou P (2017) Physicochemical and colloidal aspects of food matrix effects on gastrointestinal fate of ingested inorganic nanoparticles. Adv Colloid Interface Sci 246:165-180. . https://doi.org/10.1016/J.CIS.2017.05.010
7. Mudunkotuwa IA, Rupasinghe T, Wu C-, Grassian VH (2012) Dissolution of ZnO nanoparticles at circumneutral pH: A study of size effects in the presence and absence of citric acid. Langmuir 28:396-403.
8. Xu Z, Grassian VH (2017) Bovine serum albumin adsorption on TiO2 nanoparticle surfaces: Effects of pH and coadsorption of phosphate on protein-surface interactions and protein tructure. J Phys Chem C 121:21763-21771.
